# Changes in hepato-renal gene expression in microminipigs following a single exposure to a mixture of perfluoroalkyl acids

**DOI:** 10.1371/journal.pone.0210110

**Published:** 2019-01-04

**Authors:** Akiko Sakuma, Haruyo Wasada Ochi, Miyako Yoshioka, Noriko Yamanaka, Mitsutaka Ikezawa, Keerthi S. Guruge

**Affiliations:** 1 Miyagi Prefectural Sendai Livestock Hygiene Service Center, Anyoji, Miyagino, Sendai, Miyagi, Japan; 2 Cellular Biology Laboratory, Graduate School of Agricultural Science, Tohoku University, Aramaki Aza Aoba, Aoba, Sendai, Miyagi, Japan; 3 Kumamoto Prefectural Central Livestock Hygiene Service Center, Jyounan-mahi Shizume, Minami, Kumamoto, Kumamoto, Japan; 4 National Institute of Animal Health, National Agriculture and Food Research Organization, Kannondai, Tsukuba, Ibaraki, Japan; 5 Graduate School of Life and Environmental Sciences, Osaka Prefecture University, Izumisano, Osaka, Japan; 6 Centre for Crop Health, University of Southern Queensland, Toowoomba Campus, Queensland, Australia; National Institutes of Health, UNITED STATES

## Abstract

It is evident that some perfluoroalkyl acids (PFAAs), a group of globally dispersed pollutants, have long biological half-lives in humans and farm animals. However, the effects of PFAAs in domestic animals have not been fully elucidated. The present study investigated how exposure to a single dose of a mixture of 10 PFAAs influenced hepatic and renal gene expression and histopathology, as well as plasma clinical biochemistry, in microminipigs (MMPigs) over 21 days. In animals treated with PFAAs, the mRNA expression of twelve genes related to fatty acid metabolism was upregulated in the kidney, while only few of these genes were induced in the liver. The expression of several kidney injury-associated genes such as, IGFBP1, IGFBP6, GCLC X2, GCLC X3, MSGT1, OLR1 was upregulated in the kidney. Interestingly, the expression of IGFBP-genes was differentially altered in the liver and kidney. Our findings thus identified hepato-renal gene expression changes in MMPigs that were associated with various molecular pathways including peroxisome proliferation, lipid metabolism, kidney injury, and apoptosis. Furthermore, serum HDL levels were significantly decreased following exposure to PFAAs, whereas no significant histopathological changes were detected, as compared to the vehicle group. Taken together, the present study provided the first indication that a single exposure to a mixture of PFAAs can produce changes in MMPig renal gene expression that were observed three weeks post exposure, suggesting that more attention should be paid to the kidney as a primary target organ of PFAAs.

## Introduction

For several decades, perfluoroalkyl acids (PFAAs) have been used in a wide range of domestic and industrial applications because of their excellent physical and chemical properties. Consequently, residues of numerous PFAAs have been detected at various concentrations in almost every environmental compartment around the world. For instance, they have been detected in human blood around the globe [1–7]. This ubiquitous distribution, environmental persistence, and potential human health concerns led to the listing of perfluorooctane sulfonate (PFOS) as a Persistent Organic Pollutant (POP) by the Stockholm Convention in 2009, within Annex B (Restriction). Guideline concentrations of PFOS and perfluorooctanoic acid (PFOA) have also been stipulated for food and drinking water [8,9] to restrict human consumption of these compounds.

PFAAs have been detected in livestock animals [10,11]. In beef cattle, PFOS mostly concentrates in the liver, followed by the kidney, which showed the longest elimination half-life of 385 days; the PFOS half-life in muscle was 165 days [12]. In pigs, the whole animal elimination half-lives are 634 days for PFOS and 236 days for PFOA [13]. These time periods are much longer than the commercial pig rearing period because these animals are normally slaughtered at approximately 180 days old within the pork industry. In chickens, the blood half-lives of PFOA and PFOS were 4.6 days and 125 days, respectively, and high concentrations of these chemicals were found in the kidney and liver, respectively [14]. Sex- and species-specific renal transporter proteins may play an important role in the renal clearance and resorption of PFAAs in humans and other animal species [15, 16]. Epidemiological studies further suggest that elevated human blood levels of PFOA and PFOS may be associated with kidney disease in adults and kidney dysfunction in children [17–18]. This suggests that the kidney is both important for the elimination of PFAAs and also a target organ for the effects of these compounds. Exposure experiments in animals reported that PFAAs elicited transcript signatures that included many known gene targets of peroxisome proliferator-activated receptor alpha (PPARα), indicating effects on lipid metabolism [19]. Humans exposed to PFOA and PFOS showed altered blood expression of genes involved in cholesterol metabolism [20]. Nevertheless, the organ-specific effects of PFAAs on the transcriptome have not been elucidated for pigs.

Microminipigs (MMPigs) are the smallest pig species. These animals provide an attractive model for toxicological studies because they are easy to handle and cost-effective. We previously reported the blood elimination half-lives of 10 PFAAs, which are frequently detected in the environment, in MMPigs, in addition to their organ-specific accumulation patterns, following the administration of a single oral dose of mixed PFAAs [21]. However, the biological effects of exposure to PFAAs have not been evaluated in MMPigs. Moreover, we still lack an understanding of how PFAAs exposure regulates genes in farm animals. Therefore, the present study investigated the histopathological and transcription-level effects of a single PFAAs dose on MMPig hepatic and renal tissues, in addition to conducting analyses of their plasma clinical biochemistry. Previous animal exposure experiments have focused on hepatic, rather than renal, effects, even though substantial levels of PFAAs accumulate in the kidney, where they exhibit longer biological half-lives than those observed in the liver. To address this, we studied the expression of several genes associated with kidney injury, in addition to those involved in fatty acid metabolism.

## Materials and methods

### Animals and sample storage

The present study used frozen liver and kidney tissues from five sexually mature female MMPigs (Fuji Micra Inc., Shizuoka, Japan) aged 5.6-8.5 months and weighed 9–14 kg; these tissues were collected during a previous study [21]. In brief, the animals were separated into a control group (n = 2; C1 and C2) and an exposure group (n = 3; E1, E2, and E3). The animals were individually kept in stainless steel open-stall-type cages before and during the exposure. The exposure group was treated orally with a single gelatin capsule (100% pig) containing 3 mg kg^-1^ body weight of a nominal mixture of each 10 PFAAs (30 mg kg^-1^ body weight PFAAs) mixed with sugar powder. These compounds were perfluorobutanoic acid (PFBA), perfluoropentanoic acid (PFPeA), perfluorohexanoic acid (PFHxA), perfluoroheptanoic acid (PFHpA), PFOA, perfluorononanoic acid (PFNA), perfluorononanoic acid (PFDA), perfluorodecanoic acid (PFUnDA), perfluorododecanoic acid (PFDoDA), and PFOS. Blood samples were collected before and after exposure, at 0, 1, 2, 4, 11, 15, and 21 days, and plasma samples were stored at -20°C until use. At 20 days, food was removed from the animals over 24 h before euthanasia under deep anesthesia with sodium pentobarbital, and the animals were necropsied to collect tissue samples. Details of the exposure protocol and the concentrations of PFAAs in blood and tissue samples have been reported previously [21]. This experiment was conducted according to the guidelines for animal experiments of the National Institute of Animal Health (NIAH, Tsukuba, Japan). The protocol was approved by the committee on the ethics of animal experiments of the NIAH (Protocol Number: 11–076).

Tissues (liver and kidney) were stored in RNA*later* (Ambion Inc., Texas, USA) at -20°C for the evaluation of gene expression and were also fixed in 10% neutral buffered formalin and embedded in paraffin for histological examination. Paraffin sections were stained with hematoxylin and eosin.

### Clinical biochemical measurements in plasma

The plasma levels of aspartate aminotransferase (AST), alkaline phosphatase (ALP), γ-glutamyl transpeptidase (γ-GTP), total bilirubin (T-Bil), alanine aminotransferase (ALT), total protein (TP), blood urea nitrogen (BUN), albumin (Alb), uric acid (UA), creatinine (Cre), total cholesterol (T-Chol), triglycerides (TG), high-density lipoprotein cholesterol (HDL), lactate dehydrogenase (LDH) in plasma were investigated by SPOTCHEMTMEZ SP-4430 (ARKRAY, Inc., Kyoto, Japan). Low-density lipoprotein (LDL) was estimated by LDL(mg/dl) = (T-Chol)–(HDL + TG/5).

### RNA isolation and analysis by reverse transcription-quantitative PCR

RNA was extracted from approximately 30 mg of each tissue using the Quick Gene RNA tissue kit S II (Kurabo Industries Ltd, Osaka, Japan) in accordance with the manufacturer’s protocol and including DNase treatment.

Reverse transcription was carried out using total RNA (50 ng/μL) with PrimeScript RT Master Mix (Perfect Real Time) (Takara Bio Inc., Shiga, Japan) in accordance with the manufacturer’s instructions. Real-time PCR reactions were carried out using SYBR Premix Ex Taq II (Tli RNaseH Plus) (Takara Bio Inc., Shiga, Japan) using the protocol provided by the manufacturer. The real-time PCR conditions were 95°C for 30 sec, followed by 40 cycles of 95°C for 5 sec and 60°C for 30 sec. A melting curve analysis of the amplicons was then conducted at 95°C for 15sec, 55°C for 60 sec followed by 55–95°C at a heating rate of 0.5°C/sec. Genes were analyzed using a Mx3000P real-time qPCR system (Agilent Technologies, La Jolla, CA). The primer details are shown in Table 1 [22].

**Table 1:** Primer sequences for real-time qPCR.

Gene Symbol	Forward Primer	Reverse Primer	Reference
CYP4A21	TTTTCCCGCTTGAGGAGTGC	ACTCGGTCTGTGTGTTGATGGA	This study
CAT	GTCCTGAGTCTCTGCATCAGGTT	GTTCATGTGCCTGTGTCCATCT	[22]
PYGL	GAAATTCTCCAGTGACCGAACAA	AGGTCGGAAGGCTCCATGT	This study
SCD	CTCTGGGCGTTTGCCTACTA	GGGGCAGTCGAGCTTTGTAA	[22]
FADS1	CCGCGACACAACTACCACAA	CTTGGACTGGTACTCTATACCATGCTT	[22]
FADS2	CGCGACCTTGATTTAGTGCG	AGTTCTTGGTGCGATCCTGG	[22]
ACADM	TGGCAATGAAAGTTGACCTAGCTA	TCGGCGACCAGAATCAATC	[22]
EHHADH	CCTCTGGAGCATCCTGGAAA	CAAGCCGAGAATGCCAACA	[22]
CPT1A	GGACATCCCGGAGGAGTGT	CACGTCGTCCGCCAGAA	[22]
ACOX	CTTTGTGCAGCGAGGACATC	CAAGGTGGGCAGGAACATG	[22]
PPARα	GGCACTGAACATCGAATGTAGAAT	TGCAACCTTCACAGGCATGA	[22]
ABCD3	CGCGCTGGTGCACTCAT	TGGCGATGGCAAGACTGTT	[22]
IGFBP1	CACAGCAAACAGTGCGAGAC	CATTTGGGGTCCCCTCTGAC	This study
IGFBP2	CCAGGAGTTCTGACATGCGT	CATCTCCAGCTGGGCATCTC	This study
IGFBP3	ACAAGAAAAAGCAGTGCCGC	CCGTACTTATCCACGCACCA	This study
IGFBP4	CAGCCCTCTGACAAGGACGAG	GCTCCGGTCTCGGATCTTGG	This study
IGFBP5	CTGTGACCGCAAGGGATTCT	GGCAGCTTCATCCCGTACTT	This study
IGFBP6	CCCTCGGGGGAGAATCCTAA	GAGGGAGTGGTAGAGGTCCC	This study
GCLC X2	TCCAGGTAACGTTCCAAGCC	TCAATGGGACAATTGGGCAG	This study
GCLC X3	ACATCTACCACGCCGTCAAG	GAGAGAGAACCAACCTCGTCG	This study
MGST1	CTCCTGCTCAGATCCACAATTC	ATAGGAGGCAAAGGCCATGAA	This study
OLR1	GCGGCAAACTTTTCAGGTCC	AGCAGTTCTCCCGGCTTTTT	This study
MFGE8	TTCGCCTTCTCCGGTGACTTC	TGGGGATCCTGGTCCAACAA	This study
LCN2	AAACCACGCTTTACGGGAGG	GACTTGGCAAAGCGGACAAA	This study
GAPDH	CCAGGTTGTGTCCTGTGACT	GCTTGACGAAGTGGTCGTTG	This study

The PCR reactions were carried out using primers for cytochrome P4504A21 (CYP4A21), catalase (CAT), glycogen phosphorylase L (PYGL), stearoyl-CoA desaturase (SCD), fatty acid desaturase 1 (FADS1), fatty acid desaturase 2 (FADS2), acyl-CoA dehydrogenase (ACADM), enoyl-CoA hydratase (EHHADH), carnitine palmitoyltransferase 1A (CPT1A), acyl-CoA oxidase 1 (ACOX), peroxisome proliferator activated receptor alpha (PPARα), and ATP binding cassette subfamily D member 3 (ABCD3). The primer sets for kidney injury biomarkers, including insulin like growth factor binding proteins (IGFBPs; 1, 2, 3, 4, 5, 6), glutamate-cysteine ligase catalytic subunit (GCLC), transcript variant X2 and X3, microsomal glutathione S-transferase 1 (MGST1), oxidized low density lipoprotein receptor 1 (OLR1), milk fat globule-EGF factor 8 protein (MFGE8), and lipocalin 2 (LCN2), were additionally used. The level of each gene transcript was normalized to the expression of glyceraldehyde-3-phosphate dehydrogenase (GAPDH), and calculated using the 2^−△△Ct^ method, where ΔΔCt was the difference between the target gene cycle threshold [Ct]—GAPDH Ct of the exposure and control groups.

### Statistics

The biochemical data were presented as the mean ± standard error of the mean, and were analyzed by F-test and either Student’s t-test or Welch’s t-test. The statistical significance threshold was p = 0.05.

## Results

### Physiological status and the accumulation of PFAAs

No exposure-related clinical signs were observed during the 21-day study period [21]. The mean total concentration (± standard error) of PFAAs in the liver, blood and kidney was 75677±22660, 39307±6700 and 23075±3286, respectively [21].

### Clinical biochemistry in plasma

**Fig 1:**
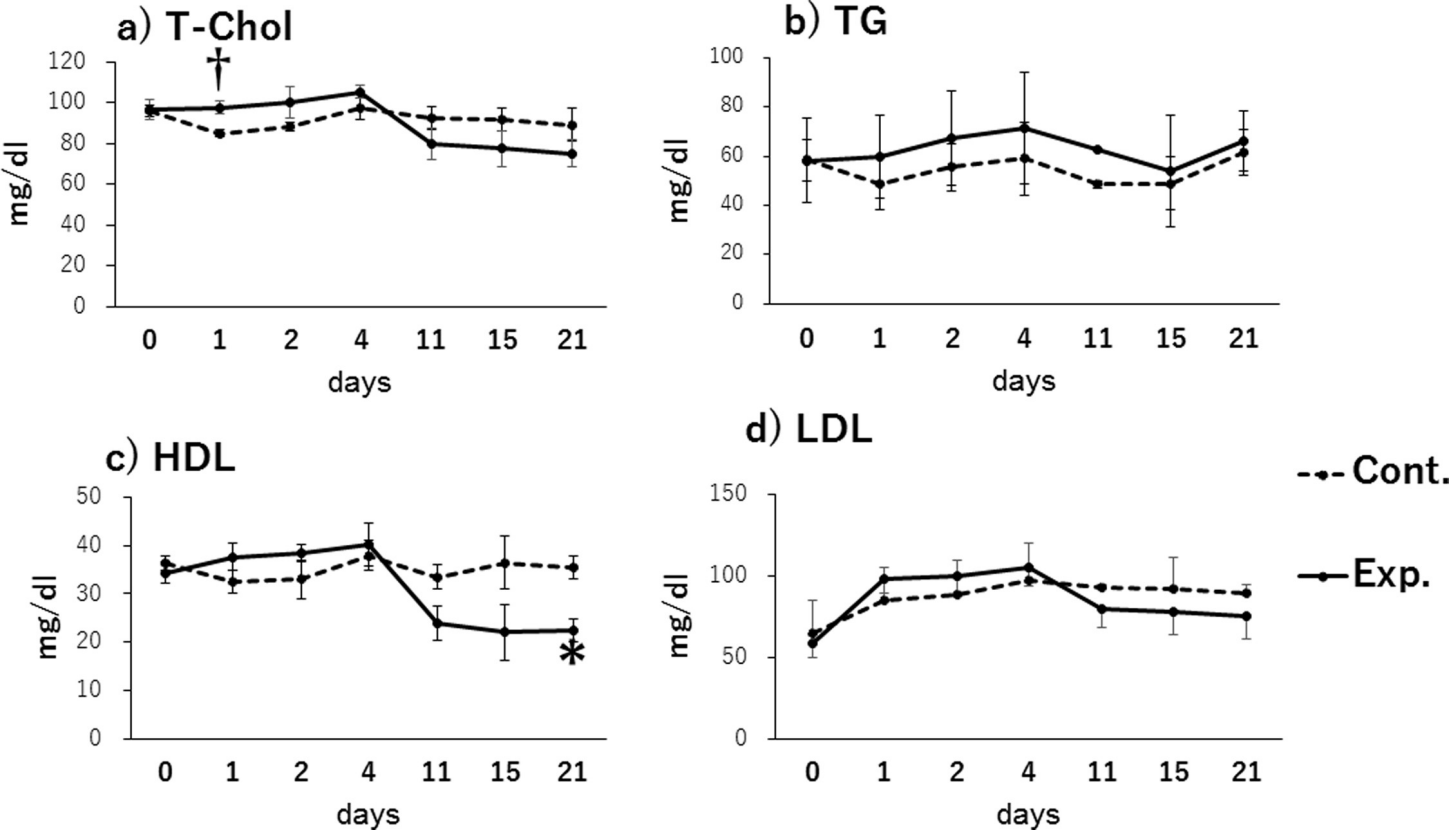
Levels of lipid metabolism markers in plasma. a) Total cholesterol (T-Chol); b) triglycerides (TG); c) high-density lipoprotein (HDL); d) Estimated Low-density lipoprotein (LDL). †p < 0.1, *p < 0.05 for the comparison between the control group (Cont.) and the exposure group (Exp.).

Biochemical analyses of plasma samples revealed significant differences in lipid metabolism. The HDL level was significantly lower in the exposure group, as compared to the control group, at day 21 post exposure (p < 0.05, Fig 1). The T-Chol level in the exposure group tended to be higher than that of the control group on post exposure day 1 (p < 0.1), but no subsequent differences were observed during the study period (Fig 1). The ALP activity in the exposure group tended to be lower than that observed in the control group (S1 Fig), and this difference was significant at 15 days post exposure (exposure group: 370.7 ± 22.4 IU/L, control group: 676.5 ± 59.5 IU/L; p < 0.05). However, no differences were observed in the other plasma biochemistry parameters examined in the exposure and control groups (e. g. TG and LDL in Fig 1).

### Histopathology

**Fig 2:**
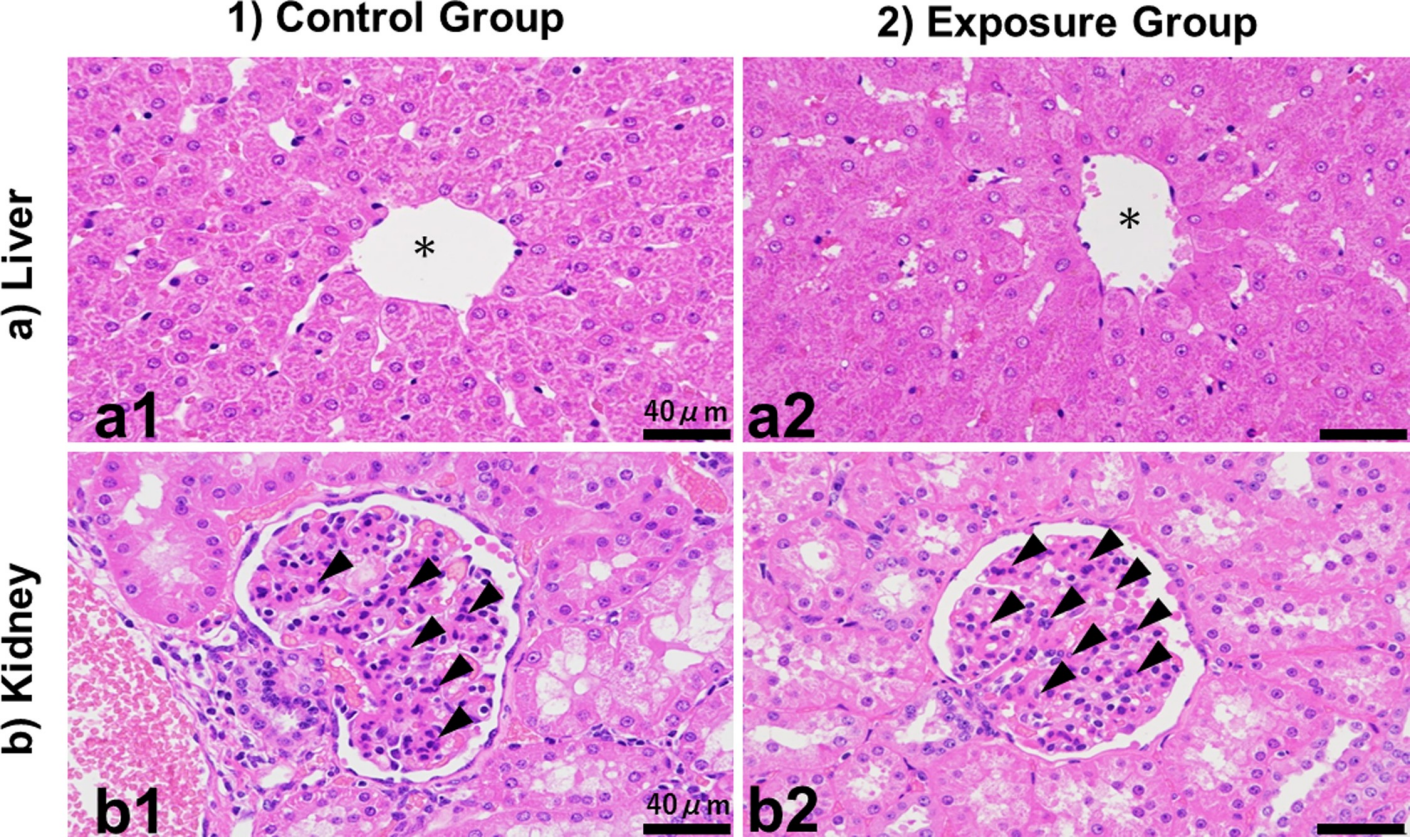
Results of histopathological examination. **1) Control group, 2) Exposure group.** a) Sections of area around the central vein in liver. * shows a central vein, b): Sections of glomerulus in kidney. Arrows show mesangial cells and area., a2: Livers in exposure group have slightly low levels of glycogen granules compared to those in control group (a1). b1: and b2: Mesangial cells in glomerulus slightly increased in both control group and exposure group. Magnification x 400.

Histopathological examination revealed a slight decrease in the liver glycogen content in the exposure group, as compared to the control animals, but no other significant differences were detected in the liver or kidney tissues (Fig 2).

### Metabolism-associated gene expression

**Fig 3:**
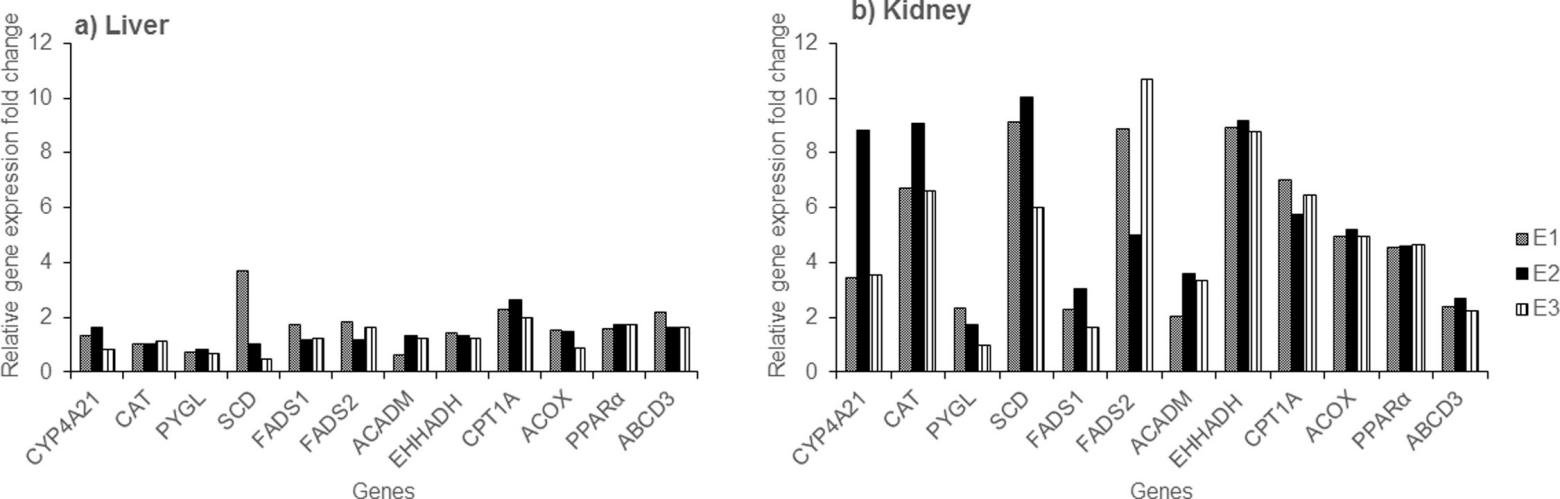
**Relative mRNA expression in a) liver; and b) kidney tissues, expressed as fold change in the exposed group (n = 3; E1, E2, and E3).** The genes analyzed were associated with fatty acid metabolism.

The mRNA levels of metabolism-associated genes are shown in Fig 3. In the liver, the gene expression of CPT1A, PPARα, and ABCD3 was increased (>1.5-fold) in the exposed MMPigs, as compared to the control group (CPT1A: 2.3 ± 0.3-fold; PPARα: 1.7 ± 0.1-fold; ABCD3: 1.8 ± 0.3-fold) (mean ± standard deviation); FADS2 expression was also increased by 1.5 ± 0.3-fold. Interestingly, the mRNA levels of all metabolism-associated genes were upregulated in the kidneys of the exposure group, as compared to the control group: CYP4A21 (5.3 ± 3.1-fold), CAT (7.5 ± 1.4-fold), PYGL (1.7 ± 0.7-fold), SCD (8.4 ± 2.1-fold), FADS1 (2.3 ± 0.7-fold), FADS2 (8.2 ± 2.9-fold), ACADM (3.0 ± 0.8-fold), EHHADH (8.9 ± 0.2-fold), CPT1A (6.4 ± 0.6-fold), ACOX (5.0 ± 0.2-fold), PPARα (4.6 ± 0.1-fold), and ABCD3 (2.4 ± 0.2-fold).

### Kidney injury-associated gene expression

**Fig 4:**
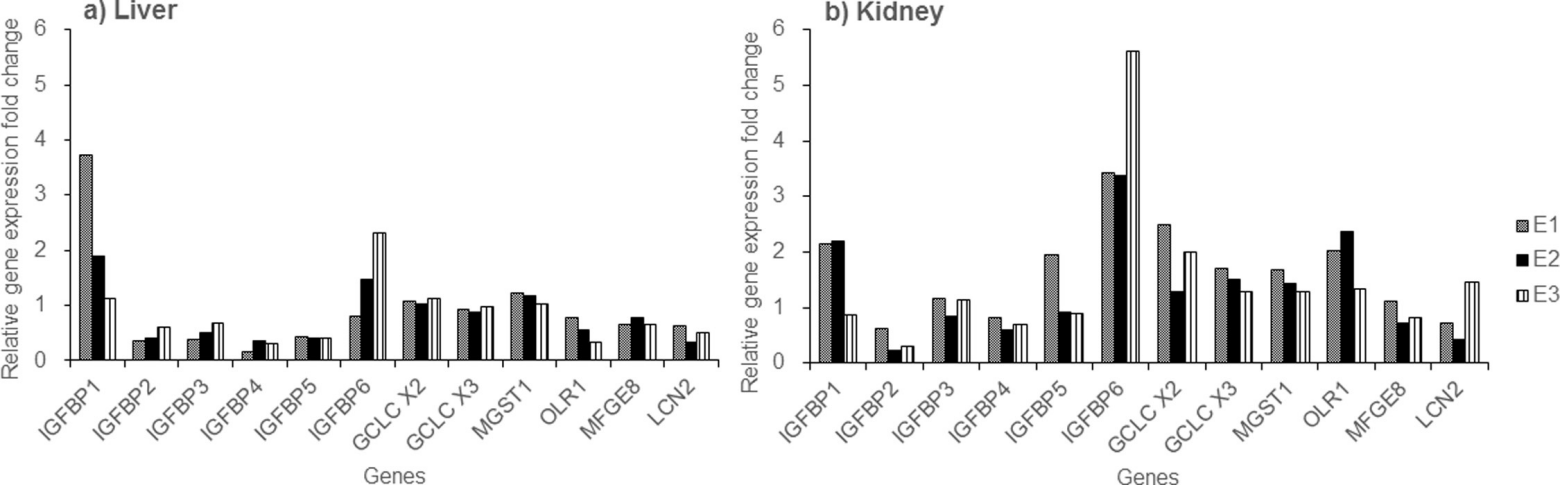
Relative mRNA expression of genes associated with kidney injury in a) liver and b) kidney, expressed as fold change in the exposed group (n = 3; E1, E2, and E3).

The mRNA levels of kidney injury-associated biomarkers are shown in Fig 4. In the liver, the expression of IGFBP1 was 2.3 ± 1.3-fold higher in the exposure group, as compared to the control group. In contrast, the expression of IGFBP2 (0.5 ± 0.1-fold), IGFBP3 (0.5 ± 0.1-fold), IGFBP4 (0.3 ± 0.1-fold), and IGFBP5 (0.4 ± 0.0-fold) was lower in the exposure group than in the control group. Moreover, the expression of OLR1 (0.6 ± 0.2-fold), MFGE8 (0.7 ± 0.1-fold), and LCN2 (0.5 ± 0.2-fold) also tended to be lower in exposed MMPigs, as compared to control animals.

In the kidney, IGFBP6 (4.1 ± 1.3-fold) showed the greatest upregulation of the IGFBP family members; IGFBP1 (1.7 ± 0.8-fold) tended to increase slightly, while IGFBP2 tended to decrease (0.4 ± 0.2-fold), as compared to the control group. The expression levels of GCLC X2 (1.9 ± 0.6-fold), GCLC X3 (1.5 ± 0.2-fold), MSGT1 (1.5 ± 0.2-fold), and OLR1 (1.9 ± 0.5-fold) exhibited slight upregulation, as compared to the control group. No clear changes in the expression levels of IGFBP3, 4, 5, MFGE8, or LCN2 were observed in the exposure group, as compared to the control group (Fig 4).

## Discussion

In the current study, we investigated changes in plasma clinical biochemistry, histopathology, and the expression of genes related to fat metabolism and kidney injury in MMPigs following the administration of a single dose of a mixture of PFAAs. We employed only 5 animals (control = 2 and treated = 3) to minimize the number of experimental animals. Although hyperplasia, vacuole formation, and atrophy have been reported in rats fed with PFOS [9], we only observed a slight decrease in the liver glycogen content in MMPigs (Fig 2), 21 days after exposure. In an earlier study, the liver glycogen content was reduced in pigs that had been fasted for 24 h or treated with clofibric acid (a potent peroxisome proliferator) for one week [22]. Hence, the minor effects on liver glycogen observed in the present study may have been influenced by exposure to PFAAs. The PFOS and PFOA concentrations in blood of MMPigs in this study were within the range of those reported in sera for occupationally exposed workers [1, 23].

Our observation of significantly lower plasma HDL concentrations in MMPigs exposed to PFAAs is consistent with previous sub-chronic PFOS exposure studies conducted in monkeys [24] and chickens [14]. The reason for the significant increase in total cholesterol level observed on day 1 post exposure is not known; however, a reduction relative to the controls was observed after 21 days. Seacat et al. [24] reported that although the serum levels of T-Chol and HDL varied during the exposure period, both of these levels were reduced by the end of the sub-chronic PFOS exposure period in monkeys, consistent with the present observation in MMPigs.

Exposure to PFAAs has been reported to interfere with a large number of PPARα-dependent and -independent genes involved in fatty acid metabolism in rodents [25–27]. For instant, ACADM and CYP4A14 were upregulated in wild-type mice while ACOX1 and EHHADH were upregulated in both wild-type and PPARα-null mice exposed to PFOS [26]. Nevertheless, gene-level effects on pigs exposed to PFAAs have not been documented. We noted that the average kidney/liver concentration ratio of PFAAs was 0.305 in these MMPigs [21]. However, the expression of genes related to fatty acid metabolism were highly upregulated in the kidney, as compared to the liver; this upregulation was disproportionate to the accumulation of PFAAs. These findings suggested that PPARα and genes related to its oxidant pathway can be upregulated in the liver, and also in other important organs such as the kidneys, in pigs.

PPARα can be upregulated in the pig liver by peroxisome proliferators, and also by fasting. The expression of genes responsible for PPARα-mediated fatty acid metabolism such as CPT1A, ABCD3, ACOX, ACADM, and EHHADH was significantly or moderately increased in the pig liver in response to both fasting and clofibric acid treatment, while SCD expression was downregulated in fasted pigs, as compared to those receiving a normal diet [22]. In the present study, the expression of PPARα, CPT1A and ABCD3 was upregulated in the liver and kidney of the exposure group, as compared to the control group. In addition, the kidney expression of ACOX, ACADM, and EHHADH also increased in the group exposed to PFAAs. Nevertheless, the duration of fasting may also have influenced these observations to some extent because food was removed from all animals 24 h before euthanasia, and the control group necropsy was carried out before that of the exposure group [21]. Moreover, we found an increased hepato-renal expression of FADS2 in the exposure group, as compared to the control group (Fig 3). In addition, CYP4A, CAT, PYGL, SCD, and FADS1 expression was upregulated in the kidney in the exposure group, as compared to the control group (Fig 3). It was previously reported that expression of CYP4A, CAT, PYGL, SCD, FADS1, and FADS2 genes was significantly increased by exposure to a peroxisome proliferator, but not by fasting, in pigs [22]. Collectively, these data suggest that PPARα-mediated fatty acid metabolism was extensively elevated in MMPigs treated with PFAAs.

Epidemiological studies also suggested that kidney function may be associated with the blood levels of PFAAs [28]. As reported previously, higher levels of PFAAs in serum were linked with chronic kidney disease in domestic cats [29]. Hence, we analyzed twelve genes that were known to be associated with kidney function and injury. Expression of IGFBP1, IGFBP6, GCLC X2, GCLC X3 (GCLCs), MGST1, and OLR1 were upregulated in the kidney following exposure to PFAAs, as compared to the control group (Fig 4). The expression levels of IGFBPs, GCLCs, MGST1, and OLR1 genes are used as biomarkers of kidney injury [30–34]. Moreover, the growth hormone-insulin-like growth factor- (GH-IGF) axis plays a role in the maintenance of renal function and in the pathogenesis and progression of chronic kidney disease [32]. IGFBPs not only enhance the effects of IGFs, but also have IGF-independent activities; for instance, IGFBP1 and IGFBP6 levels increase in chronic renal failure [30]. The levels of IGFBP6 are particularly high in children with chronic renal failure, and correlate inversely with the glomerular filtration rate [35]. In addition, GCLCs, MGST1, and OLR1 can be employed as markers of oxidative stress [31, 34, 36]. GCLC mediates the synthesis of glutathione S-transferase and thus plays an important role in the intracellular antioxidant defense system [37–38]. MGST1 is activated by oxidative stress and neutralizes lipid peroxides, as well as conjugating other reactive intermediates to glutathione [39]. Our present data suggest that the expression of these genes was upregulated due to oxidative stress, which occurred as a result of the PFAAs retained in the MMPigs, even 21 days post-exposure. LCN2, a neutrophil gelatinase-associated lipocalin, is a small glycosylated protein that is regarded as a biomarker of acute kidney injury and is thought to associate with chronic kidney disease progression in humans [40–43]. In addition, LCN2 is overexpressed by mesangial cells in the damaged kidney [40, 44]. Our histological analyses indicated that both control MMPig pigs and those exposed to PFAAs had mesangial lesions (Fig 2). However, this lesion might reflect age-related glomerulonephritis, as these animals were approximately 6 months old [45]. We did not observe any upregulation of LCN2 expression; however, as indicated above, upregulation of several other injury markers was observed in the absence of any histological evidence of kidney damage. Hence, our data suggested that transcriptional changes were observed in the kidney 3 weeks after a single exposure of MMPigs to a mixture of PFAAs, without any significant nephropathy.

The IGFs and IGFBPs play an important role in cell growth and differentiation [46]. The present study identified differential alterations in the expression of IGFBPs in the liver and kidney. The expression of IGFBP1 increased in both the liver (2.3 ± 1.3-fold) and kidney (1.7 ± 0.8-fold), IGFBP6 expression only increased in the kidney (4.1 ± 1.3-fold), while reduced expression of IGFBP2 was observed in the liver (0.5-fold average) and kidney (< 0.5-fold average), and the expression of IGFBP4 and IGFBP5 was decreased in the liver (< 0.5-fold in average) (Fig 4). On the other hand, kidney expression of the oxidative stress biomarker, OLR1, was upregulated (1.9-fold average) while the serum level of HDL, which has antioxidant properties, was downregulated (Figs 1 and 4). Oxidative stress can lead to apoptosis, which is a process that inhibits tumor development. IGFBP2, 3, 4, and 5 are related to apoptosis, and IGFBP3 is known to have proapoptotic effects [30, 47]. We found that the expression of MFGE8, which is also related to apoptosis, and IGFBP3 was slightly decreased in the livers of the exposure group, as compared to the control group (MFGE8: 0.7 ± 0.1-fold, IGFBP3: 0.5 ± 0.1-fold, Fig 4). Taken together, these data suggest that exposure to PFAAs may link to the inhibition of apoptosis pathway in MMPigs.

**Fig 5:**
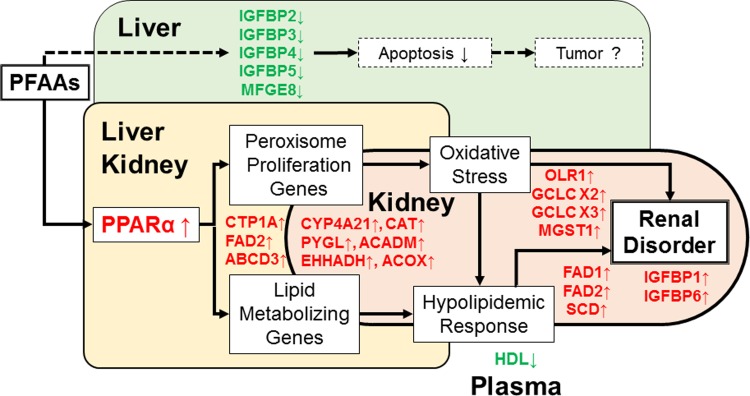
Hypothesized mode of action of PFAAs in MMPigs. The lines indicate major changes in gene expression and the downstream phenotypic responses noted in this study. The dashed lines indicate potential connections that remain to be verified.

Fig 5 shows the hypothesized mode of action of PFAAs in organs with special reference to the kidney of MMPigs, based on the observations made in this study. Our findings revealed that MMPigs exposed to PFAAs showed hepato-renal changes in the transcription of genes that were associated with cell proliferation, peroxisome proliferation, lipid metabolism, kidney injury, and apoptosis. Specifically, the present study revealed that a single exposure to a mixture of PFAAs was associated with changes in kidney gene expression at 21 days post exposure, in the absence of significant histological lesions. Nevertheless, further studies including microarray analysis are required to elucidate the molecular pathways involved in the potential adverse effects triggered by the exposure of large animals to PFAAs.

## Supporting information

S1 Fig Alkaline phosphatase (ALP) levels in plasma. †p < 0.1, *p < 0.05 for the comparison between the control group (Cont.) and exposure group (Exp.).
(DOCX)
