# Supplementary material for: Changes in hepato-renal gene expression in microminipigs following a single exposure to a mixture of perfluoroalkyl acids
Source: PLoS One. 2019 Jan 4;14(1):e0210110. doi: 10.1371/journal.pone.0210110 (PMC6319762; doi:10.1371/journal.pone.0210110)
Supplement: S1 Fig — †p < 0.1, *p < 0.05 for the comparison between the control group (Cont.) and exposure group (Exp.). (DOCX) [file pone.0210110.s001.docx]

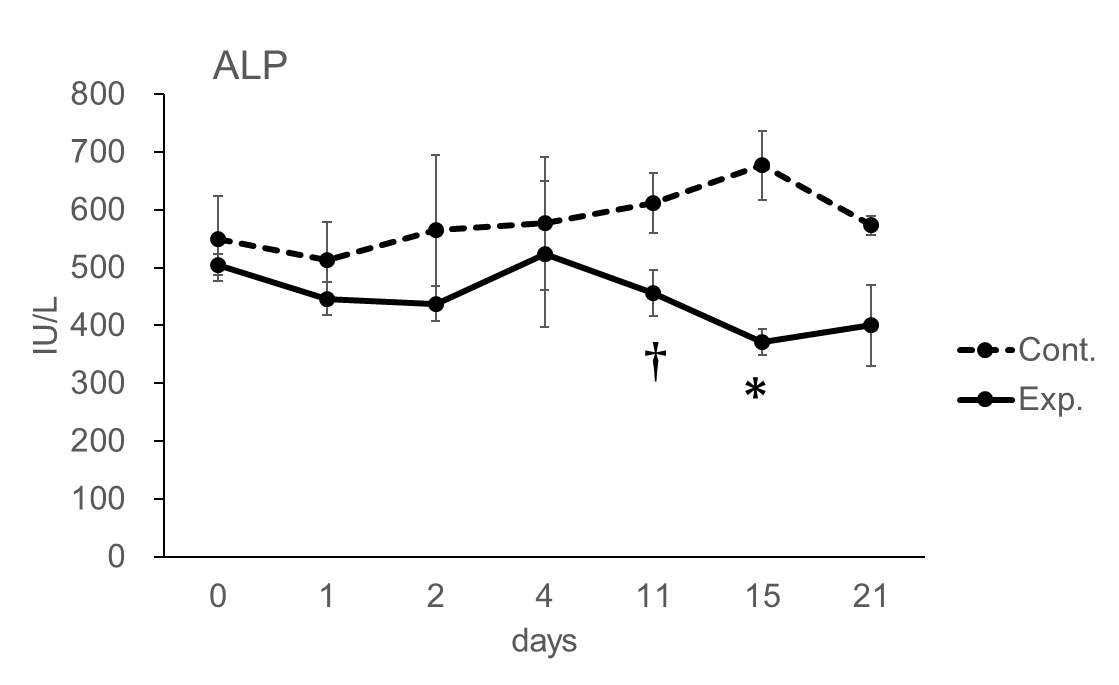


Supplementary Figure 1. Alkaline phosphatase (ALP) levels in plasma. †p < 0.1, *p < 0.05 for the comparison between the control group (Cont.) and exposure group (Exp.).
